# Relationships among social support, healthy lifestyle beliefs, physical literacy, and enjoyment of physical activity: a moderated mediation model

**DOI:** 10.3389/fpubh.2025.1617124

**Published:** 2025-08-04

**Authors:** Mehmet Akif Yücekaya, Sinan Uğraş, Ahmet Enes Sagin, Arif Çetin, Teodora-Mihaela Iconomescu, Laurențiu-Gabriel Talaghir

**Affiliations:** ^1^Department of Sports Management, School of Physical Education and Sports, Dicle University, Diyarbakır, Türkiye; ^2^Department of Physical Education and Sports Teaching, Faculty of Sport Sciences, Çanakkale Onsekiz Mart University, Çanakkale, Türkiye; ^3^Department of Coaching Education, Faculty of Sport Sciences, Bartin University, Bartýn, Türkiye; ^4^Department of Sports Management, Faculty of Sport Sciences, Marmara University, Kadıköy, Türkiye; ^5^Department of Sport Games and Physical Education, Faculty of Physical Education and Sport, Dunarea de Jos University, Galați, Romania; ^6^Department of Individual Sport and Physical Therapy, Faculty of Physical Education and Sport, Dunarea de Jos University, Galați, Romania

**Keywords:** social support, physical literacy, healthy lifestyle beliefs, physical activity, adolescents

## Abstract

**Objective:**

This study aims to examine the relationships between social support, physical literacy, and healthy lifestyle beliefs in adolescents and to investigate the moderating effect of enjoyment of physical activities on these relationships.

**Method:**

The study was conducted with the participation of 1,349 adolescents. The ages of the participants ranged between 10 and 14 years, and 49.6% were male (*n* = 669) and 50.4% were female (*n* = 680). Data were collected using the Healthy Lifestyle Belief Scale for Adolescents to measure adolescents' healthy lifestyle beliefs, the Perceived Physical Literacy Scale for Adolescents (PPLSA) to determine their physical literacy levels, the Social Support Scale in Physical Activities to assess the perception of social support, and the Enjoyment of Physical Activity Scale to measure the level of enjoyment of physical activities. The study tested the moderated mediation model for its association among the variables.

**Results:**

The model developed in this study found social support in PA predicted health lifestyle beliefs, positively, and significantly. Social support and perceptions of a healthy lifestyle are moderated by physical literacy, research shows. A further analysis also found that the relationship between physical literacy and social support is moderated by enjoyment of physical activities. However, the association between social support and healthy lifestyle beliefs and between physical literacy and healthy lifestyle beliefs were not appreciably influenced by enjoyment in physical activity. The findings indicated that social support has a positive and significant effect on physical literacy and attitudes toward healthy lifestyle. Physical literacy was identified as a central contributor to improving adolescents' health habits by mediating the relationship between social support and conceptions of healthy living. However, the enjoyment of fitness did not matter in some situations, the study found. These results further illustrate the importance of promoting social support and physical literacy instruction to promote a wellness lifestyle adoption by adolescents. The possible long-term effects of feeling good about exercising in various settings, and for health-related habits, should receive more scrutiny.

## Introduction

Physical activities (PAs) are crucial for adolescents to acquire healthy living habits so that they obtain the health benefits that can be maintained over a lifetime. World Health Organization (WHO) recommendations for physical activity and sedentary behavior for children and young people suggest that young people (5–17 years) should engage in at least 60 min of moderate to vigorous intensity physical activity each day. However, 80% of adolescents worldwide fail to reach this level (1). This low physical activity habit is associated with increased body fatness, risk of cardiovascular disease and psychosocial concerns, and proven to have a negative influence on individuals' learning, emotional wellbeing and social insertion (2, 3). It is widely agreed that exercise is known to be beneficial for physical, mental, and psychosocial health (4, 5). Nevertheless, the literature supports the need to further explore the social and individual determinants of PA behaviors in this age-group. Physical inactivity is a global public health issue that has been defined as such by WHO, which, for its part, calls for the development and implementation of national policies to address the problem (1).

**Figure 1:**
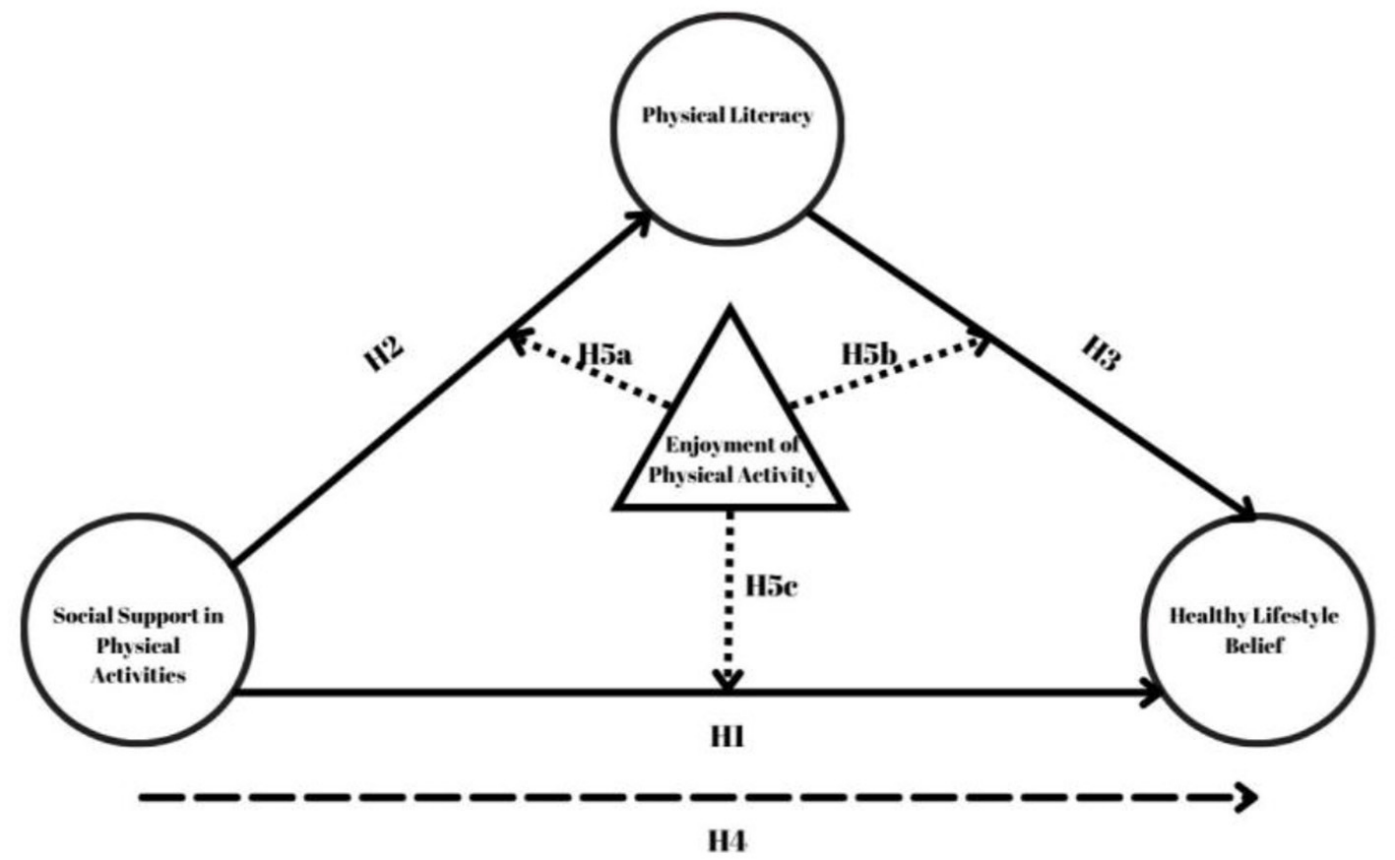
Research hypothesis.

In this respect, social support is one of the primary determinants of adolescents' physical activity (6, 7). Research shows that social support from family, peers, and teachers can increase physical activity levels and strengthen adolescents' healthy lifestyle beliefs (8, 9). Healthy lifestyle beliefs refer to individuals' cognitive assessments, attitudes, and perceptions of self-efficacy regarding behaviors that support physical, mental, and emotional wellbeing. These beliefs encompass not only regular physical activity but also health-promoting lifestyle elements such as balanced nutrition, stress management, adequate sleep, and avoidance of harmful habits such as smoking or excessive screen use (10, 11). Physical activity does not represent all these beliefs while physical activity is an significant component of healthy lifestyle beliefs. However, social support decreases the feelings of loneliness of adolescents, increasing their self-confidence and adherence and motivation to perform exercise, making their contribution quite important the process's success (6). Nevertheless, the literature still has many gap about the ways that social support affects adolescents' physically active habits. Indeed, the interplay among the social support with individual constructs, including physical literacy, and healthy lifestyle beliefs has yet to be established. There is also the appealing concept of physical literacy. Physical literacy is a multi-dimensional concept comprising the knowledge, skills, motivation, and confidence to engage and demonstrate competence in physical activity throughout the lifespan. This idea enhances physical performance and influences people's values and attitudes toward physical activity. In particular, adolescence is an important time in which to develop physical literacy; the behaviors learned during adolescence typically persist into adulthood (12). Furthermore, by promoting personal self-efficacy for physical activity (13), physical literacy may help individuals to overcome barriers and adopt regular participation more easily. The positive effect during PA is one of the strongest predictors for long-term regular participation in PA (14). Fun in physical activity had made more repeatable the behavior by offering a positive experience to the people while doing physical activity (15). Perceived physical activity enjoyment plays a major role, particularly among adolescents in increasing physical activity participation (16). This feeling may be part of the process of establishing long-term habits by enhancing people's attitudes and behaviors toward PA. The current study seeks to examine the social- and individual-level correlates of adolescent PA participation. Examining the influence of social support, physical literacy, healthy lifestyle beliefs and enjoyment of physical activity on individuals' physical activity behaviors is an attempt to address a critical gap in the literature. As adolescence is a key life stage at which life-long healthy habits are formed, this research conceptualized the ways in which social support may influence on the engagement in physical literate activity participation, maintain physical literacy and its associated perceptions of a healthy lifestyle as synonymous with being physically literate. Furthermore, the mediating role of PA enjoyment and the differential moderating role of PA enjoyment on these associations have enriched related theoretical implications and applications. It is anticipated that the findings from the study will add to theoretical knowledge and facilitate the development of intervention strategies to target young people's physical activity. In this context, the following hypotheses were formulated and presented in Figure 1:

H1: Social support for physical activity positively and significantly predicts healthy lifestyle beliefs.
H2: Social support in physical activities positively and significantly predicts physical literacy.
H3: Physical literacy positively and significantly predicts healthy lifestyle beliefs.
H4: Physical literacy mediates the relationship between social support in physical activities and healthy lifestyle beliefs.
H5a: Enjoyment of physical activity moderates the relationship between social support and healthy lifestyle beliefs.
H5b: Enjoyment of physical activity moderates the relationship between social support and physical literacy.
H5c: Enjoyment of physical activity moderates the relationship between physical literacy and healthy lifestyle beliefs.

### Social support and healthy lifestyle beliefs in physical activities

Social support contributes to the development of health behaviors in adolescence. Support from family, friends and teachers is known to enhance participation in PA and enhance peoples' belief in EHW (6, 17). Families' demonstration of healthy behavior motivates adolescents to engage in these behaviors; family beliefs about physical activity are critical to the development of life-long health habits (18, 19). Moreover, emotional support and trust from parents enhance people's motivation to participate in physical activity and make them feel that they can do it.

Peer support is another important social resource in shaping health attitudes and behaviors. Adolescents participate more in physical activity and adopt healthy behaviors more easily with positive feedback from their social environment (20, 21). However, since peer influences do not always create a positive result on individuals, adolescents may also turn to negative habits with the influence of social norms, so the role of supportive social environments becomes more important in this process (22).

It is also stated that social support mechanisms have a motivational effect in the process of shaping individuals' health behaviors. Support from the social environment facilitates individuals' regular participation in physical activity and increases their self-confidence and health awareness (23, 24). Perceived social environment support and perceived individual support have a significant impact in maintaining participation in exercise. In this regard, the present study considers the effects of social support on health lifestyle beliefs and the following hypothesis, respectively:

H1: Social support in physical activities positively and significantly predicts healthy lifestyle beliefs.

### Social support and physical literacy

It is a multifaceted phenomenon that includes knowledge-based, skill-based and motivation-based dimensions to promote lifelong engagement in physical activity. Social support is an important environmental variable for the development of physical literacy, allowing individuals to become physically active on a habitual basis by increasing their self-efficacy. Family, friends and teachers are critical in determining individuals' physical activity habits (25–27).

To understand how social support related to physical literacy occurs, it is necessary to consider the concept of self-efficacy. Perception of self-efficacy is the conviction about personal ability to perform a behavior believed to be a factor affecting behavior. The sense of belonging to a social group is one of the resources that can strengthen it. For instance, people perceive others' supportive PA-relevant behaviors, which support their confidence about PA participation; behavioral modeling of parents in healthy lifestyles facilitates their offspring to develop similar attitudes toward health (25, 28). At an educational level, the social support domains provided by physical education are correlated with parental influence on motor competence. Positive learning environments enable all students to refine their motor skills and adopt appropriate attitudes toward physical activity. This physical education contribution broadens the concept physical literacy through developing the personal as well as the social abilities of the children (29, 30). The influence of social support on physical literacy extends beyond confidence. Greater exposure to social interaction enhances social skills necessary for being physically active. Peer support and encouragement are also an indispensable source of motivation in this procedure. Encouragement from peers leads to a positive attitude regarding physical activity (31).

It would be important to further investigate the influence of social support on physical literacy dynamics within the relationship described here. In this present study, the relationship between social support and physical literacy was investigated and the hypotheses were developed as follows:

H2: Social support in physical activities positively and significantly predicts physical literacy.

### Literacy and healthy lifestyle beliefs

Physical literacy has been defined as a multi-dimensional construct that includes the motivation, confidence, physical competence, knowledge, understanding and desire to be active for life. This concept has shaped the physical activity assumptions, as well as the health behavior, self-efficacy, and health in general of the individual. For example, a 5–11-year-old with high levels of physical literacy will have children in whom to establish good physical lifestyle habits, including regular participation in physical activity. These findings extend to adults as well (26, 32).

Physical Literacy is confounding health behaviors is particularly evident among the young. Formal education interventions (e.g., PE programs) can reinforce student beliefs about health and enhance physical literacy. The programs also develop the physical ability of the student and help them to normalize good living habits. For example, children given higher body competence engagement scores are more likely to participate in physical activity regularly and normalize healthy behavior (33). This highlights the importance of physical literacy in education.

In addition, health literacy, in particular e-health literacy, can play a role in how people perceive healthy lifestyle issues. Research shows that individuals with high health literacy are more likely to engage in health-promoting behaviors, including good nutrition and regular physical activity. For example, in one study that targeted college students, a high level of e-health literacy was associated with a health-promoting lifestyle (34). Therefore, we may argue that physical literacy is centrally involved in the formation of health-promoting lifestyle attitudes. The overlap of the 2 literacies (health and physical) would also demonstrate how people adopt health behavior for life. The current work has the following hypothesis:

H3: Physical literacy positively and significantly predicts healthy lifestyle beliefs.

### Mediating effects between social support, healthy lifestyle beliefs, and physical literacy

The relationship between social support and healthy lifestyle beliefs is complex and multifaceted. In the literature, there are findings that physical literacy may play a role as a mediating factor in explaining the relationship between social support and healthy lifestyle beliefs. Family support and peer support, facilitate the learning of physical literacy skills by increasing confidence and motivation in individuals to engage in physical activity. For example, Kouvonen et al. (35) noted that the presence of positive social connections can contribute to levels of motivation in the lives of individuals to engage in healthy activities which include exercise on a regular basis and help to develop their physical literacy levels. Similarly, development of physical literacy is key to establishing healthy lifestyle attitudes. People who are more skilful in sports may be more open to use the long-term health benefits related to these behaviors. Liu et al. (36) speculated on the social support in reducing the negative effects of low health literacy on healthier behaviors. Meng et al. (37) also reveals that physical literacy gains predict psychological wellbeing, and that those with a higher level of physical literacy are more likely to adopt and sustain health promoting lifestyle affections.

The mediating role of physical literacy is especially robust in the mediated relationship between social support and beliefs of a healthy lifestyle. Chen et al. (13) suggested that health literacy was yet another possible mediator of the relationship between social capital and health actions. This result may indicate that health literacy could lead to desirable health beliefs and behaviors, especially when supported by social support. Likewise, physical literacy can be both a skill set and a factor for social connection so that we can all make healthy choices.

Overall, the association of social support and physical literacy with the belief in a healthy lifestyle highlights the importance of environments that promote physical literacy development. The current study proposed to mediate between social support for physical activities and health lifestyle beliefs. This hypothesis was formulated as follows:

H4: Physical literacy mediates the relationship between social support for physical activities and healthy lifestyle beliefs.

### The regulatory role of enjoyment of physical activity

The enjoyment experienced during PA is one of the primary motivators that influence people's routine participation in PA. This refers to the good experiences people have when engaging in physical activity, both in terms of the immediate effect of the exercise and the opportunities to repeat the exercise. Findings imply that liking physical activity may enhance the social support-attitude toward physical activity relationship. Hence, people who enjoy physical activity are more likely to keep doing it even with social support for instance. Conversely, people who lack liking in physical activities may lose out on access to social support when it comes to participation in physical activities, this creates a decline in the participation of physical activities (38, 39).

The enjoyment of physical activities can act as a moderator of the associations between social support, physical literacy and beliefs toward healthy lifestyles. Pleasure raises the frequency of physical activity exposure and aids the process of converting these acts into habits. Research reveals that those with strong physical activity preferences are more likely to develop positive attitudes and self-efficacy for physical activities. This may reinforce a physical active life course to them (40, 41). Nonetheless, those whom do not appreciate doing physical activity might face the challenge of feeling more reluctant to take part in physical activity regardless of the support they may receive and may not sustain them (42).

One might speculate that liking to partake in physical activity acts as a moderator variable in the associations between social support, physical literacy and healthy lifestyle beliefs. For instance, physical activities enjoyment was linked to positive psychosocial outcomes that enhance the continuation of activities by promoting individual's motivation and self efficacy (43, 44). Moreover, interventions to enhance physical activity enjoyment have been associated with substantially higher rates of participation and adherence to physical activity programs, particularly in younger and older populations (38, 39).

The results in the literature have shown that liking activities could strengthen the effects of both the social support and the physical literacy that lead to healthy lifestyle beliefs. Conversely, it is suggested that social support and physical literacy may have little influence upon those low in enjoyment levels, findings that could have an impact upon the sustainability of positive health behaviors. In this easily constricted regulatory domain the role of enjoyment of physical activity is viewed as one important focus of the present investigation. The following hypotheses were developed in accordance with this information:

H5a: Enjoyment of physical activity has a moderating role in the relationship between social support and healthy lifestyle.
H5b: Enjoyment of physical activity has a moderating role in the relationship between social support and physical literacy.
H5c: Enjoyment of physical activity has a moderating role in the relationship between physical literacy and healthy lifestyle beliefs.

## Materials and methods

### Research group

Before starting the research, permission was obtained from the Dicle University Social and Human Sciences Ethics Committee with the approval number 795939. We calculated the sample size using G^*^Power software for the moderated mediation analysis. In the calculation using the multiple regression model, we found that the effect size was moderate (*f*^2^ = 0.15), with an alpha level of 0.05, a power of 0.90, and a total of 4 independent variables (the independent variable, mediator variable, moderator variable, and interaction term). According to these parameters, the minimum required sample size was found to be 634 people. The participants in the study consisted of a total of 1,349 students attending three state secondary schools in three different provinces of Turkey. The participants were in grades 5, 6, 7, and 8, and the classes included in the study were determined using random sampling. The questionnaires were administered in the classroom environment during class hours and under the supervision of the researchers. This method ensured that the students answered the questions independently and carefully. Each questionnaire took an average of 20–25 min to complete. Prior to the application, students were informed about voluntary participation and confidentiality principles, and written parental consent was obtained. A total of 1,400 students were surveyed, and 51 forms containing incomplete or invalid data were excluded from the analysis; thus, 1,349 valid surveys were used in the analysis. While 49.6% of the participants were male (*n* = 669), 50.4% were female (*n* = 680). 25.6% (*n* = 346) of the participants were 5th graders, 21.6% (*n* = 291) were 6th graders, 26.2% (*n* = 354) were 7th graders, and finally 26.5% (*n* = 358) were 8th graders. 23.9% (*n* = 323) of the participants stated that they participated in extracurricular sportive activities. Data was collected from five different schools with different economic environments in Adyaman, Türkiye. All students attended 2 h of compulsory physical education classes per week, and 23.9% (329) of students were found to participate in 6 h of extracurricular sports activities per week. The study included students with an average age of 11.73 (SD = 1.13) and calculated their average body mass index as 17.92 (SD = 2.87). Confirmatory factor analysis was performed to test the construct validity of each data collection tool specific to this research. We also performed Cronbach's alpha analyses.

### Data collection tools

#### Healthy lifestyle belief scale for adolescents

The original scale, which was developed to measure adolescents' awareness of healthy lifestyle behaviors and their self-efficacy to change these behaviors, was adapted from Melnyk's previous studies (10, 45) and developed by Kelly et al. (11). Kudubeş and Bektaş (46) adapted the scale into Turkish. The scale has a five-point Likert structure and consists of 16 items. Each item is scored from 1 = strongly disagree to 5 = strongly agree. A minimum of 16 and a maximum of 80 points can be obtained from the scale. The increase in the score obtained from the scale indicates that adolescents' healthy lifestyle beliefs increase. CFA analysis was conducted to test the construct validity of the scale in this study. According to the results of the CFA analysis, it was determined that the item factor loadings ranged between β = 0.445 and β = 0.675, and the CFA fit values were *X*^2^ = 502/d*f* = 104, *p* = < 0.001, CFI = 0.915, TLI = 0.902, IFI = 0.915, GFI = 0.985, SRM*R* = 0.039, and RMSEA = 0.065. Cronbach's alpha value was calculated as 0.885. According to these results, it can be stated that the Healthy Lifestyle Belief Scale has valid and reliable values (47).

#### Perceived Physical Literacy Scale for Adolescents (PPLSA)

The scale developed to determine the perceived physical literacy levels in adolescents was adapted into Turkish by Yilmaz and Kabak (48). It is a measurement tool consisting of 3 dimensions: “Knowledge and Understanding,” “Self-expression and Communication with Others,” and “Sense of Self and Self-confidence,” and a total of 9 items. The higher the average scores obtained from the sub-dimensions, the higher the physical literacy perceptions of the individuals. The measurement tool is a 5-point Likert scale and is scored from strongly disagree = 1 to strongly agree = 5. According to the results of the DFA analysis conducted to test the structure of the data collection tool for this study, the item factor loadings ranged between β = 0.320 and β = 0.636, while the DFA fit values were *X*^2^ = 106/d*f* = 25, *p* = < 0.001, CFI = 0.961, TLI = 0.943, IFI = 0.961, GFI = 0.998, SRM*R* = 0.029, and RMSEA = 0.049. The Cronbach alpha value was calculated as 0.735. These values show that the data collection tool is within acceptable limits (47).

#### Social support scale in physical activities

The scale developed by Farias Junior et al. (49) to determine the social support perceived by adolescents in physical activities is a 4-point scale consisting of 10 items and two sub-dimensions: 0 = Never, 3 = Always. The scale was adapted to Turkish culture by Küçükibiş and ve Eskiler (50). The 5 items evaluating the sub-dimensions of the family support and peer support scale contain the same statements. The increase in the average score obtained from the scale means that the perceived social support increases. According to the results of the DFA analysis conducted to test the structure of the data collection tool for this study, it was determined that the item factor loads ranged between β = 0.603 and β = 0.703 and the DFA fit values were *X*^2^=177/d*f* = 33, *p* = < 0.001, CFI = 0.965, TLI = 0.952, IFI = 0.965, GFI = 0.989, SRM*R* = 0.029, and RMSEA = 0.057. The Cronbach alpha value was calculated as 0.735. These values show that the data collection tool is within acceptable limits (47, 51).

#### Enjoyment of physical activity scale

The scale developed by Mullen et al. (52) to determine the level of enjoyment that affects participation in and maintenance of physical activities was adapted into Turkish by Özkurt et al. (53). The scale has a 7-point Likert structure and consists of a single dimension and 8 items. It is scored as Strongly Disagree = 1 to Strongly Agree = 7, and it is understood that as the average value increases, the level of enjoyment of physical activities increases. According to the results of the DFA analysis conducted to test the structure of the data collection tool for this study, it was determined that the item factor loads ranged between β = 0.612 and β = 0.729, and the DFA fit values were *X*^2^ = 151/d*f* = 19, *p* = < 0.001, CFI = 0.967, TLI = 0.951, IFI = 0.967, GFI = 0.995, SRM*R* = 0.029, and RMSEA = 0.072. The Cronbach alpha value was calculated as 0.864. These values show that the data collection tool is within acceptable limits (47).

### Data analysis

Data analysis was performed in the Jamovi 1.2.16 statistical program. To determine the normality assumption of the data, skewness and kurtosis values were taken as criteria to determine whether the data were normally distributed (47). To test the construct validity of the data collection tools in this study, confirmatory factor analysis was conducted separately for each of them. In the CFA analysis, *X*^2^/d*f* , CFI, TLI, IFI, GFI, SRMR, and RMSEA values were considered to test the structure (47). Pearson correlation analysis was used to determine the relationships between social support in physical activities, physical literacy, healthy lifestyle beliefs, and enjoyment of physical activity. A simple mediating analysis was performed to determine the results of social support in physical activities as the independent variable, healthy lifestyle beliefs as the dependent variable, and physical literacy as the mediating variable. For the moderated mediation analysis, enjoyment of physical activity was included as a moderator variable in the Jamovi GLM section. With a 5,000-sample size and 95% confidence interval, the bootstrapping method was used to evaluate the indirect effect. According to the confidence interval value, when the lower (BootLLCI) and upper (BootULCI) confidence interval values corresponding to the indirect effect do not contain zero (0), the indirect effect is considered to be significant and the presence of a mediation effect is understood (54).

## Results

Descriptive statistics and bivariate correlations among the study variables were initially analyzed to explore general trends and associations. Table 1 presents descriptive statistical values for the key variables addressed in the study and the correlation levels between these variables.

**Table 1:** Mean, standard deviation, skewness, kurtosis and Pearson correlation results of the variables.

**Variables**	**SSPA**	**HLB**	**PL**	**EPA**	**Mean**	**SD**	**Skewness**	**Kurtosis**
SSPA	–				1.73	0.716	−0.408	−0.302
HLB	0.488^***^	–			3.93	0.767	−0.725	0.500
PL	0.465^***^	0.636^***^	–		3.90	0.729	0.0341	4.28
EPA	0.383^***^	0.589^***^	0.556^***^	–	5.85	1.036	−0.450	1.76

^*^p < 0.05, ^**^p < 0.01, ^***^p < 0.001.

SSPA, Social Support in Physical Activities; HLB, Healthy Lifestyle Belief; PL, Physical Literacy; EPA, Enjoyment of Physical Activity.

Correlation coefficients and their significance levels (^*^p < 0.05, ^**^p < 0.01, ^***^p < 0.001) are shown in Table 1. The mean for social support for physical activities (*M* = 1.73, SD = 0.716), the mean for healthy lifestyle belief (*M* = 3.93, SD = 0.767), the mean for physical literacy (*M* = 3.90, SD = 0.729), and the mean for physical activity enjoyment (*M* = 5.85, SD = 1.036) were found. According to Pearson correlation analysis results, positive and significant (*r* = 0.488, *p* < 0.001), positive and significant (*r* = 0.465, *p* < 0.001), and positive and significant (*r* = 0.383, *p* < 0.001) relationships were found between social support for physical activities and healthy lifestyle belief, between social support for physical activities and physical literacy, and between social support for physical activities and enjoyment of physical activity. There was a positive and significant relationship between healthy lifestyle beliefs and physical literacy (*r* = 0.636, *p* < 0.001) and a positive and significant relationship between healthy lifestyle beliefs and enjoyment of physical activity (*r* = 0.589, *p* < 0.001). Finally, a positive and significant (*r* = 0.556, *p* < 0.001) relationship was found between physical literacy and enjoyment of physical activity.

Table 2 shows the direct, indirect and total effects of the PL variable on the relationship between SSPA and HLB.

**Table 2:** Mediation effects.

**Type**	**Effect**	***β***	**S.E**.	***Z*-value**	***p***	**95% confidence interval**	
						**Lower**	**Upper**
Indirect	SSPA → PL → HLB	0.243	0.0203	12.78	< 0.001	0219	0.243
Direct	SSPA → HLB	0.245	0.0284	9.23	< 0.001	0.207	0.318
	SSPA → PL	0.465	0.0277	17.07	< 0.001	0.419	0.527
	PL → SSPA	0.522	0.0417	13.16	< 0.001	0.466	0.629
Total	SSPA → HLB	0.488	0.0255	20.50	< 0.001	0.472	0.572

SSPA, Social Support in Physical Activities; HLB, Healthy Lifestyle Belief; PL, Physical Literacy.

**Figure 2:**
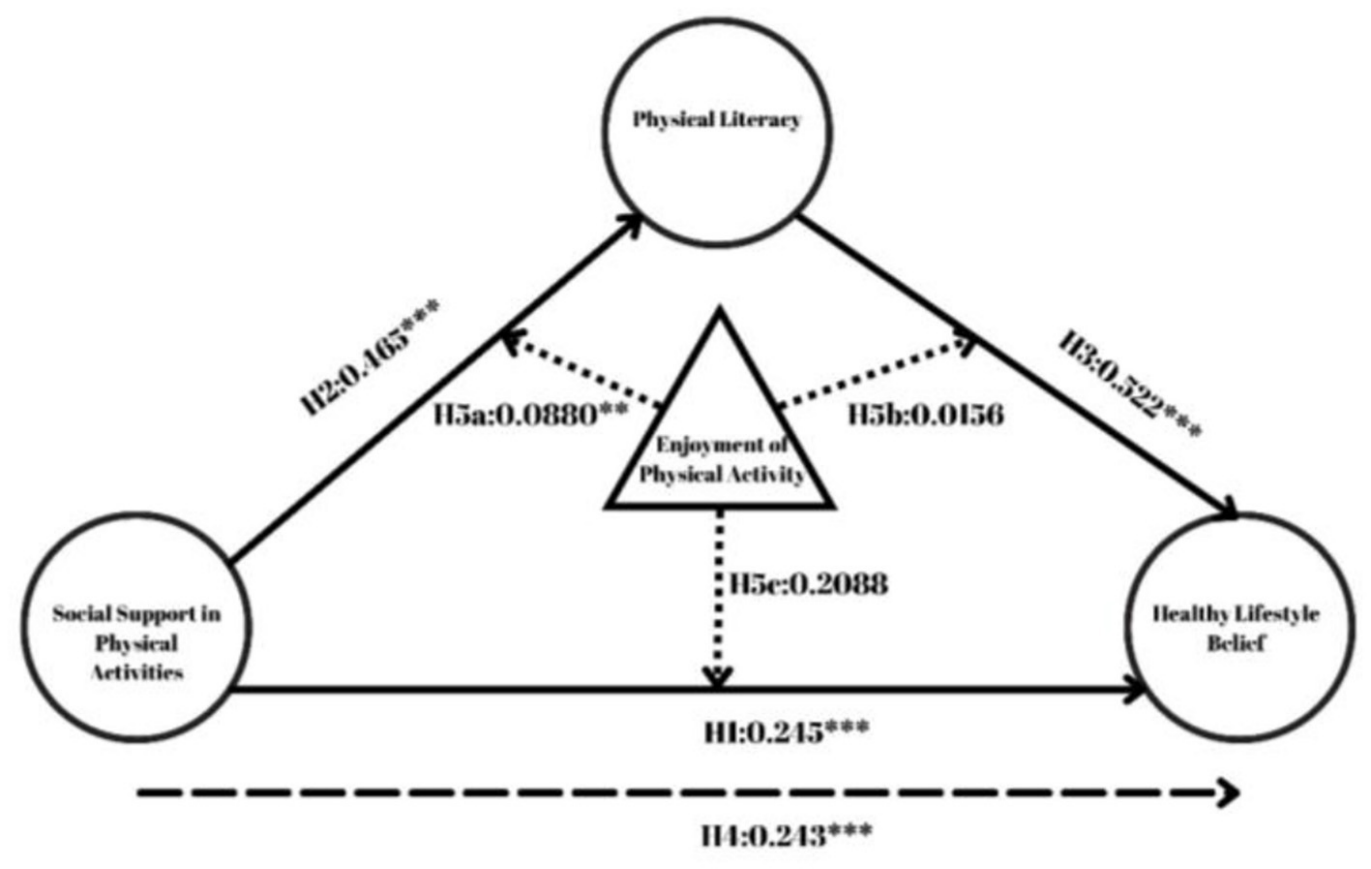
Hypothesis results. *p < 0.05, **p < 0.01, ***p < 0.001.

According to the mediation analysis results of the study, it was found that social support in physical activities (SSPA) positively and significantly predicted healthy lifestyle beliefs (HLB) through physical literacy (PL) (β = 0.243, *z* = 12.78, *p* < 0.001, CI [0.219, 0.243]). In addition, social support in physical activities directly predicted healthy lifestyle beliefs in a positive and significant way (β = 0.245, *z* = 9.23, *p* < 0.001, CI [0.207, 0.318]). In addition, it was found that social support in physical activities positively and significantly predicted physical literacy (β = 0.465, *z* = 17.07, *p* < 0.001, CI [0.419, 0.527]). Similarly, physical literacy was found to have a positive and significant relationship with healthy lifestyle beliefs (β = 0.522, *z* = 13.16, *p* < 0.001, CI [0.466, 0.629]). Finally, when the total effects were analyzed, it was found that perceived social support in physical activities predicted a healthy lifestyle positively and significantly in total (β = 0.488, *z* = 20.50, *p* < 0.001, CI [0.472, 0.572]). The results of the analysis are shown in Figure 2.

Table 3 presents the findings regarding the regulatory effects of the EPA variable on the relationships between SSPA and PL and HLB.

**Table 3:** Moderation effects.

**Interaction**	**β**	**SE**	**Lower**	**Upper**	***z***	***p***
SSPA EPA → PL	0.0880	0.0200	0.0134	0.0917	2.654	0.008
SSPA EPA → HLB	0.0156	0.0226	−0.0329	0.0556	0.436	0.663
EPA PL → HLB	0.2088	0.0252	−0.0179	0.0810	1.331	0.183

SSPA, Social Support in Physical Activities; HLB, Healthy Lifestyle Belief; PL, Physical Literacy; EPA, Enjoyment of Physical Activity.

**Figure 3:**
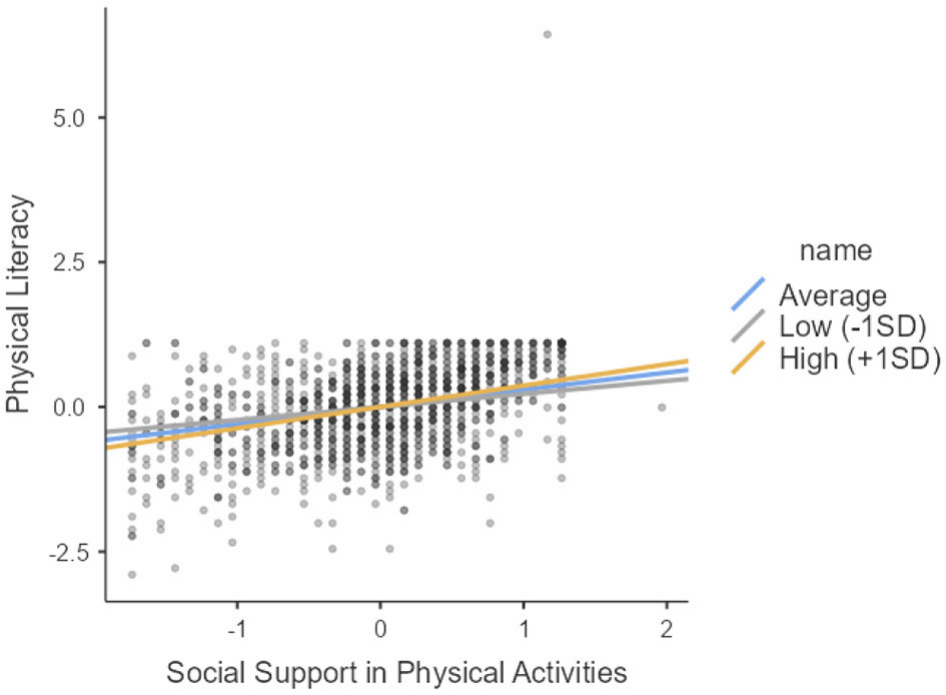
Simple slope plot.

According to the moderation analysis results of the study, the effect of the interaction of social support for physical activities (SSPA) and enjoyment of physical activity (EPA) on physical literacy (PL) was found to be positive and significant (β = 0.088, SE=0.020, *z* = 2.654, *p* = 0.008, CI [0.0134, 0.0917]). However, the effect of the interaction of SSPA and EPA on healthy lifestyle beliefs (HLB) was not significant (β = 0.0156, SE=0.0226, *z* = 0.436, *p* = 0.663, CI [−0.0329, 0.0556]). Similarly, the effect of EPA-PL interaction on HLB was not significant (β = 0.2088, SE=0.0252, *z* = 1.331, *p* = 0.183, CI [−0.0179, 0.0810]). The results of the moderated mediation analysis are shown in Figure 3. The simple slope graph of the regulatory role of enjoying physical activity in the relationship between social support in physical activities and physical literacy, which was found to be significant as a result of moderated mediation analysis, is shown in Figure 3.

## Discussion

Our study examined the relationships between social support, physical literacy, healthy lifestyle beliefs, and enjoyment of physical activity in physical education and sport. The results show that social support strongly affects healthy lifestyle beliefs and physical literacy. In addition, physical literacy was found to affect healthy lifestyle beliefs positively and play a mediating role in the relationship between social support and healthy lifestyle beliefs. The moderating role of enjoying physical activity was found to be significant in the relationship between social support and physical literacy, but no significant moderating effect was observed in the relationships between social support and healthy lifestyle beliefs and between physical literacy and healthy lifestyle beliefs. In this section, the findings obtained are discussed in detail.

H1: Social support in physical activities positively and significantly predicts healthy lifestyle beliefs.

The findings of our study reveal that social support has a positive and significant effect on healthy lifestyle beliefs. Social support is a powerful mechanism shaping individuals' health-related attitudes and behaviors. This finding is consistent with previous studies in the literature. For example, Kelly et al. (11) found that social support has a positive relationship with healthy life beliefs and emphasized that social support facilitates individuals to adopt and maintain health behaviors. Beets et al. (55) also showed that especially parental support positively affected young people's participation in physical activity and health beliefs. In addition, Kiernan et al. (56) found that social support encourages health behaviors and increases individuals' motivation to maintain their health goals. However, De Ridder et al. (57) emphasized that healthy eating habits are not only based on individual intentions and knowledge, but also the social and physical environment are among the important determinants of these behaviors. Similarly, Ball et al. (58) drew attention to the importance of social, cultural, and environmental factors that shape individuals' health-related behaviors and emphasized the effect of support from the social environment on the maintenance of physical activity and healthy eating habits. Studies have shown that support from the social environment plays an important role in maintaining physical activity and healthy lifestyle choices. In this context, it is understood that social support is a critical factor for individuals to internalize healthy behaviors and ensure their continuity.

H2: Social support in physical activities predicts physical literacy in a positive and significant way.

The findings of our study show that social support in physical activities has a positive and significant effect on physical literacy. Social support is fundamental in helping individuals gain knowledge and skills related to physical activities. This result supports the studies in the literature. For example, Sheridan et al. (7) emphasized that social support promotes skill development and increasing participation in physical activity in young athletes. Mendonça et al. (59) stated that social support supports learning processes by facilitating seeing physical activities as a social activity. The study conducted by Castelli et al. (60) in the context of the Comprehensive School Physical Activity Program (CSPAP) revealed that the support provided by families and teachers supported individuals to gain knowledge and skills related to physical activities. These findings suggest that social support may play an important role in developing knowledge and skills, one of the basic components of physical literacy. The results of our study indicate that social support mechanisms can increase the physical literacy levels of individuals by supporting their learning and implementation processes in physical activities. In this context, the study of De Silva et al. (61) reveals that the physical activity participation rates of individuals who receive social support are significantly higher compared to individuals who do not receive support. The study also stated that in cases where family and friend support is provided together, the rates of participation in physical activity increase even more. These findings suggest that social support not only supports the acquisition of knowledge and skills in physical activities but also plays a critical role in their sustainability. The results of our study indicate that social support mechanisms can increase physical literacy levels by supporting individuals' learning and practice processes in physical activities.

H3: Physical literacy positively and significantly predicts healthy lifestyle beliefs.

The findings of our study reveal that physical literacy has a positive and significant effect on healthy lifestyle beliefs. The literature has strong evidence that physical literacy plays a critical role in shaping individuals' health awareness and behaviors. For example, Mendoza-Muñoz et al. (62) emphasized that physical literacy enhances individuals' health awareness and supports healthy life beliefs. Our study contributes to these findings, demonstrating that physical literacy reinforces individuals' health beliefs and effectively transforms these beliefs into daily habits. Similarly, Longmuir and Tremblay's (63) study, which emphasizes the self-esteem-enhancing effect of physical literacy, aligns with our findings. The self-confidence gained from physical activities facilitates individuals in transforming their beliefs about health into sustainable behaviors. Additionally, Holler et al. (64) highlighted the importance of physical literacy in adopting regular physical activity habits. Furthermore, our study reveals that physical literacy establishes a strong connection between individuals' health awareness and the behaviors that stem from this awareness. This insight allows us to understand better the fundamental role of physical literacy in developing individuals' knowledge and skills related to physical activities. The findings of Roetert et al. (65), which indicate that physical literacy encourages lifelong participation in physical activity, also resonate in this context. This study shows that physical literacy contributes positively and significantly to individuals' participation in physical activity and their health-related goals.

H4: Physical literacy has a mediating role in the relationship between social support and healthy lifestyle beliefs in Physical Education and Sport.

The study found that physical literacy mediates the relationship between social support and positive health lifestyle beliefs. Social support also influences health-related attitudes and beliefs to enhance physical literacy, knowledge, and skill in physical activities. These results agreed with the work of Caldwell et al. (66). Menescardi and Estevan (67), as well, state that social support enhances the physical literacy level of individuals through the acquisition of skills and motivation toward physical activities. Lisboa et al. (68) also found that social support from family or friends results in learning knowledge and skills about physical activities. Families reinforced active orientations by making available tools, car rides, or choices to support physical activity, while friends affect such motivation by setting social norms. Moreover, in the course of this project, we found that physical literacy strongly mediates individuals' health beliefs into action. This evidence shows the significant role of social support in learning about physical activity. Furthermore, Yang et al. (69) state physical literacy is also involved in the extent of the association between personal health-related beliefs and their role in transforming them into action. At this level, social support sustains the development of knowledge and skills and knowledge associated with the practice of physical activity. The results of this research indicate that physical literacy adds value to individuals' engagement in physical activity and pursuit of health-related aspirations.

Hypothesis H5 and Sub-Hypotheses: General Framework.

Hypothesis H5 assumes that enjoyment of physical activity may mediate the relationships between social support, physical literacy, and healthy lifestyle beliefs. This hypothesis is based on the view that enjoying physical activities may facilitate individuals interacting more effectively with these mechanisms and adopting healthy behaviors. In our research, sub-hypotheses H5a, H5b, and H5c were examined to support hypothesis H5, and it was observed that these sub-hypotheses produced different results.

H5a: Enjoyment of physical activity has a moderating role in the relationship between social support in physical activities and healthy lifestyle beliefs.

The H5a hypothesis examined the moderating role of enjoyment of physical activity on the association between social support and HLSB. This association did not significantly vary according to the degree of enjoying physical activity in our study. It might be that social support demonstrates a strong and direct impact on healthy lifestyle beliefs which suppress the effect of personal factors. Kelly et al. (11) highlighted the importance of social support as directly shaping health behavior, arguing that personal beliefs about health are largely founded on social support webs. In this regard, it has been shown that aspects of social and environmental support for behavior are influential for adolescents' PA participation, rather than internal motivations or enjoyment of the behavior. Also, Eskiler and Küçükibiş (70) found that social support has a higher significant influence over health beliefs in adolescents when compared with the level of physical activity participation. There are also related findings in a study by Dishman et al. According to the present study, the decrease of physical activity across adolescence is not solely explained by individual-level variables such as self-efficacy or enjoyment of physical activity, but it should be viewed in the light of social interaction with the environment. All of these results indicate that the non-moderating impact of the enjoyment of physical activity variable in the relation between social support and healthy lifestyle beliefs within our study can be attributed to the overpowering power of the social support construct in the context.

H5b: Enjoyment of physical activity has a moderating role in the relationship between social support in physical activities and physical literacy.

Our study revealed that enjoying physical activities significantly moderates the relationship between social support and physical literacy. Enjoying physical activities supports individuals' learning processes and skill development by enabling them to utilize social support mechanisms more effectively. These findings align with Garn et al.'s (71) research, which indicates that enjoying physical activities increases individuals' motivation for physical activity and strengthens social support mechanisms. While social support promotes participation in physical activity, the enjoyment of these activities enhances satisfaction and sustainability in this process. Similarly, Rudolf et al. (72) found that enjoying physical activities amplifies the effect of social support mechanisms on health literacy by boosting individuals' motivation to engage in physical activity. In this context, it is noteworthy how social support mechanisms facilitate individuals' knowledge and skill acquisition in physical activities and how their effectiveness can be enhanced through regulatory processes such as planning. Molloy et al. (73) emphasize that planning processes can strengthen the impact of social support. These findings indicate that social support and the processes of developing knowledge and skills related to physical activities (physical literacy) can be fortified and made more sustainable through the enjoyment of physical activities. The study by Öztürk et al. (74) further supports this process, highlighting the positive relationship between physical literacy and physical activity levels. In particular, it was revealed that enjoying physical activities increases individuals' motivation, making this process more sustainable. Our hypothesis demonstrated how enjoying physical activity regulates the relationship between social support and physical literacy by revealing the impact of these mechanisms on physical literacy.

H5c: Enjoyment of physical activity has a moderating role in the relationship between physical literacy and healthy lifestyle beliefs.

Our study revealed that enjoyment of physical activities does not significantly moderate the relationship between physical literacy and healthy lifestyle beliefs. While enjoyment of physical activities is an important factor for individuals to adopt and maintain these activities, it is noteworthy that no moderating effect was observed in this study. Our findings suggest that the direct influence of physical literacy on health beliefs may overshadow the role of regulatory mechanisms, such as enjoyment of physical activities. The literature indicates that enjoying physical activities may strengthen the connections between physical literacy and health behaviors by promoting engagement in physical activities (69). However, Buja et al. (75) emphasized that the strong impact of health literacy on physical activity may restrict the influence of regulatory mechanisms. The findings of our study suggest that the dominant role of physical literacy in shaping health beliefs may have resulted in the inability to observe the effect of regulatory factors.

The findings of our study suggest that the dominant role of physical literacy in shaping health beliefs may have resulted in the inability to observe the effect of regulatory factors. This study has revealed that social support has a significant direct effect on both physical literacy and healthy lifestyle beliefs. The findings also support the mediating role of physical literacy and emphasize the central importance of this construct. In contrast, no moderating effect of the variable “enjoyment of physical activity” was observed; this can be explained by the dominant effect of social support in these relationships, which overshadows individual factors.

## Conclusion

This study provides important insights into how social support shapes individuals' perceptions of physical literacy and healthy lifestyles. The research shows that individuals with high perceptions of social support are more likely to develop stronger physical literacy, defined as the desire to participate in physical activities throughout their lives and physical fitness. This is closely related to positive thoughts, attitudes, and behaviors regarding adopting and maintaining a healthy lifestyle. Additionally, the study identifies physical literacy as a mediating factor in the relationship between social support and its outcomes. This dual role and situation emphasize it as a factor that directly contributes to both health awareness and lifestyle choices. In addition, enjoyment of physical activity emerged as an important factor in the study. Enjoyment of physical activity also emerged as an important mechanism in this relationship. Because these individuals are more likely to enhance the development of physical literacy. Additionally, the enjoyment derived from physical activity may be influenced by age, cultural background, or type of physical activity. In this context, it is also recommended that similar studies be conducted in the future. These findings may serve as an important guide in shaping educational and public health policies aimed at encouraging lifelong participation in physical activity, particularly among adolescents.

## Limitations

While this study provides meaningful contributions to the understanding of social support and physical literacy, several limitations should be acknowledged:

- The study focused exclusively on middle school students, which may restrict the generalizability of the findings to other age groups or populations with differing demographic characteristics.
- The data collection relied on participants' self-reports, introducing the potential for response bias, which may affect the overall validity and reliability of the findings.
- Although the sample included individuals from diverse cultural backgrounds, the study did not thoroughly investigate how cultural norms influence perceptions of social support and physical literacy. This limits a deeper understanding of the cultural context within these relationships.

The data used in this study are based on participants' self-reports. Self-reports may be influenced by factors such as social desirability and recall bias, which may have a limiting effect on the validity of the results.

## Future implications

In light of these findings, the following directions are recommended for future research and practice:

- Strengthening school, family, and community-based social support initiatives can enhance physical literacy and beliefs about maintaining a healthy lifestyle.
- Future programs should integrate physical literacy's direct and mediating effects to support overall wellbeing.
- Given that the influence of social support can vary by context, educational and recreational activities should be developed to make physical engagement more enjoyable and sustainable.
- Future studies should adopt longitudinal designs to understand better how the interplay between social support, physical literacy, and healthy lifestyle beliefs evolves.

## Data Availability

The raw data supporting the conclusions of this article will be made available by the authors, without undue reservation.
